# Chronic cough and laryngeal dysfunction improve with specific treatment of cough and paradoxical vocal fold movement

**DOI:** 10.1186/1745-9974-5-4

**Published:** 2009-03-17

**Authors:** Nicole M Ryan, Anne E Vertigan, Peter G Gibson

**Affiliations:** 1 Centre for Asthma and Respiratory Diseases, School of Medicine and Public Health, The University of Newcastle, Newcastle, 2308, NSW, Australia; 2 Department of Respiratory and Sleep Medicine, Hunter Medical Research Institute, John, Hunter Hospital, Newcastle, 2310, NSW, Australia; 3 Department of Speech Pathology, John Hunter Hospital, Newcastle, 2310, NSW, Australia

## Abstract

**Rationale:**

Chronic persistent cough can be associated with laryngeal dysfunction that leads to symptoms such as dysphonia, sensory hyperresponsiveness to capsaicin, and motor dysfunction with paradoxical vocal fold movement and variable extrathoracic airflow obstruction (reduced inspiratory airflow). Successful therapy of chronic persistent cough improves symptoms and sensory hyperresponsiveness. The effects of treatment for chronic cough on laryngeal dysfunction are not known.

**Objective:**

The aim of this study was to investigate effects of therapy for chronic cough and paradoxical vocal fold movement.

**Methods:**

Adults with chronic cough (n = 24) were assessed before and after treatment for chronic persistent cough by measuring quality of life, extrathoracic airway hyperresponsiveness to hypertonic saline provocation, capsaicin cough reflex hypersensitivity and fibreoptic laryngoscopy to observe paradoxical vocal fold movement. Subjects with chronic cough were classified into those with (n = 14) or without (n = 10) paradoxical vocal fold movement based on direct observation at laryngoscopy.

**Results:**

Following treatment there was a significant improvement in cough related quality of life and cough reflex sensitivity in both groups. Subjects with chronic cough and paradoxical vocal fold movement also had additional improvements in extrathoracic airway hyperresponsiveness and paradoxical vocal fold movement. The degree of improvement in cough reflex sensitivity correlated with the improvement in extrathoracic airway hyperresponsiveness.

**Conclusion:**

Laryngeal dysfunction is common in chronic persistent cough, where it is manifest as paradoxical vocal fold movement and extrathoracic airway hyperresponsiveness. Successful treatment for chronic persistent cough leads to improvements in these features of laryngeal dysfunction.

## Background

Chronic persistent cough is responsible for a significant illness burden in the community [1]. Laryngeal problems are increasingly recognized as being part of the chronic cough syndrome, and include voice symptoms such as dysphonia [2], hyperresponsiveness of the extrathoracic airway with enhanced glottic stop reflex [3], reduced inspiratory airflow following a provocation stimulus [4-6], and paradoxical vocal fold movement (PVFM) where the vocal folds paradoxically adduct during inspiration [7,8]. Speech language therapy is effective for laryngeal dysfunction, and a randomized controlled trial has shown that speech language therapy treatment based on the approaches used in vocal cord dysfunction and hyperfunctional voice disorders is also effective in chronic cough [6]. Speech language therapy has been shown to improve symptoms [6] and voice abnormalities [9] in refractory chronic cough, however the effect on other laryngeal problems in chronic persistent cough is not known. We hypothesized that treatment of patients with chronic cough and laryngeal dysfunction would result in improvement of afferent cough reflex sensitivity and the laryngeal abnormalities of paradoxical vocal fold movement and extrathoracic airway hyperresponsiveness. The aim of this study was to investigate effects of therapy for chronic cough and paradoxical vocal fold movement.

## Methods

### Subjects

Subjects with chronic persistent cough (n = 24) were recruited from the Respiratory Ambulatory Care Service at John Hunter Hospital in Newcastle, New South Wales, Australia. Subjects were aged between 18 and 80 years with a persistent cough of more than eight weeks. They were non-smokers or ex-smokers with less than ten pack years, had no other active respiratory or cardiac disease, and were required to have a normal chest radiograph. They were classified into 2 groups based on the presence (n = 14; Cough+PVFM) or absence (n = 10; Cough alone) of PVFM observed at fibreoptic laryngoscopy. All subjects provided written informed consent for this study, which was approved by the University of Newcastle's Human Research Ethics Committee and the Hunter New England Human Research Ethics Committee.

### Study Design

Subjects attended a total of 5 visits over a period of 18 weeks. At visit 1, clinical history, current respiratory symptoms, medication use, passive smoking history and an in-house rhinitis symptoms score were recorded. A number of questionnaires were also administered and these included a cough specific quality of life questionnaire (Leicester Cough Questionnaire, (LCQ)) [10], a gastroesophageal reflux symptoms questionnaire [11], a generic quality of life questionnaire (SF36) [12] and a laryngeal dysfunction questionnaire (LDQ) [13].

All subjects were non-smokers or ex-smokers with less than 10 pack years and not exposed to current passive smoking and this was confirmed by exhaled carbon monoxide measurement [14,15]. Fractional expired nitric oxide (FENO) was also measured [16]. At visit 2 each subject underwent capsaicin cough reflex sensitivity testing (CRS) [17,18] followed by sputum induction using 4.5% saline [19]. Visit 3 included a fibreoptic laryngoscopy, followed by hypertonic saline provocation challenge (HSC) with inspiratory flow volume curve measurement [20,21] and then post-challenge laryngoscopy. The chronic cough subjects were then treated for their cough-related diagnoses (see below). Subjects returned 8 weeks after treatment to complete post treatment visits. Visit 4 repeated symptom questionnaires, FENO, CRS and sputum induction. Laryngoscopy was repeated before and after hypertonic saline provocation challenge at visit 5. Inspiratory/expiratory flow volume curves were performed before and during saline challenge, after each dose.

### Treatment Programme

A probability based diagnostic assessment approach was used [22] with the addition of induced sputum analysis to identify eosinophilic bronchitis [23], fibreoptic laryngoscopy to identify PVFM [24], and history and polysomnography to identify obstructive sleep apnea [25]. Asthma was established by doctor's diagnosis and current bronchial hyperresponsiveness and subjects were treated with inhaled corticosteroid/long-acting beta agonist combination (budesonide/eformoterol 200/6 mcg bd via Turbuhaler, AstraZeneca Sweden). Gastroesophageal reflux was suggested by a history of heartburn, dysphagia, or acid regurgitation, or an association between cough and posture or eating. Antireflux therapy included proton pump inhibitor (omeprazole 20 mg bid) and antireflux measures including advice about diet and sleeping posture. Rhinosinusitis was suggested by symptoms of nasal obstruction or sneezing, postnasal drip, nasal discharge, and when clinical or fibreoptic nasendoscopic examination of the nasopharynx and oropharynx revealed mucosal inflammation or mucopurulent secretions. In the absence of these criteria, a sinus computed tomography (CT) scan was performed if there was strong clinical suspicion of rhinosinusitis. Subjects with rhinitis received oral antihistamine (cetirizine, 10 mg od) and nasal corticosteroid spray (budesonide 128 mcg bid). Angiotensin Converting Enzyme inhibitors (ACE-I) were ceased and replaced with alternate antihypertensive medication. Subjects with eosinophilic bronchitis (induced sputum eosinophils > 3%) received inhaled corticosteroid/long-acting beta agonist combination (budesonide/eformoterol 200/6 mcg bd via turbuhaler, AstraZeneca, Sweden). Subjects with PVFM were treated with speech language therapy that was administered by a speech pathologist that involved 4 weekly sessions addressing education, vocal hygiene, cough suppression strategies, relaxed throat breathing techniques and psychoeducational counseling [6]. Obstructive sleep apnea was suggested by a history of snoring, sleep disturbance or excessive daytime somnolence, confirmed by overnight polysomnography, and treated by nasal continuous airways pressure (nCPAP).

### Clinical Methods

#### Forced Expired Nitric Oxide

Forced Expired Nitric Oxide (FENO) was measured using an on-line chemiluminescence analyser (NiOx, Aerocrine AB, Smidesvägen 12, SE-171 41 Solna, Sweden) according to published European Respiratory Society/American Thoracic Society guidelines [16]. Subjects inhaled medical-grade compressed air that contained < 2 ppb NO and then exhaled via a high expiratory resistance while targeting a mouth pressure of 20 mm Hg. This produces an expiratory flow rate of 50 mL/s (including analyser sampling rate). Exhalations were repeated until three plateau FENO values vary by < 5%. The mean of the three replicate FENO values was used.

#### Hypertonic Saline Challenge (HSC)[26]

Prior to HSC, subjects withheld bronchodilators for their duration of action and antihistamines for 48 hours. Subjects were instructed in the correct performance of inspiratory and expiratory Flow Volume Loops (FVL). The manoeuvre consisted of tidal breathing, deep inspiration to total lung capacity, forced expiration to residual volume followed by deep inspiration to total lung capacity. Hypertonic saline (4.5%) was inhaled for doubling time periods and a inspiratory-expiratory FVL was measured, in duplicate, 60 seconds after each saline dose using a KoKo K323200 Spirometer (Technipro, North Parramatta, Australia). Forced expiratory time was held constant at subsequent manoeuvres in order to ensure consistency. If the FEV_1 _fell by more than 15%, 200 μg of salbutamol was administered via a valved holding chamber (Volumatic, Allen and Hanburys, GlaxoSmithKline Australia Pty Ltd, Boronia, Australia).

#### Capsaicin Cough Reflex Sensitivity testing (CRS) [17,18]

Solutions of capsaicin (Sigma-Aldrich Co., Castle Hill, Australia) concentrations ranging from 0.98 to 500 μM were prepared daily. Subjects inhaled single breaths (from Functional Residual Capacity (FRC) to total lung capacity (TLC)) of capsaicin aerosol from a compressed air-driven nebulizer (model 646, Technipro, North Parramatta, Australia) controlled by a dosimeter (KoKo Digidoser 323200; Technipro Marketing Pty Ltd., Sydney, New South Wales, Australia). The inspiratory flow was standardized at 0.5 L/s with an inspiratory flow regulator valve. Cough counting was done for 30 s after exposure to each dose, and the investigation ended when the subject coughed five or more times in response to one dose, or received a dose of the highest concentration.

#### Fibre Optic Laryngoscopy (FOL)

Flexible fibreoptic laryngoscopy (Pentax VNL-1330, Asahi Optical Co, Tokyo, Japan) was performed at baseline and immediately after a hypertonic saline challenge [20,21]. Prior to the procedure, the nasal cavity was anesthetised with lignocaine hydrochloride 5.0% and phenylephrine 0.5% (ENT Technologies, Malvern, Victoria, Australia). The nasendoscope was then passed into the nares and positioned above the larynx. The movements of the true vocal folds were observed during tidal respiration over a period ≥2 minutes. Adduction of the vocal folds throughout the inspiratory phase and/or the beginning of expiration was considered as PVFM. These findings encompassed paradoxical glottic closure during several respiratory cycles ranging from a partial (> 50%) adduction of the true vocal folds without cordal contact to a total closure of the anterior two-third of the vocal folds. The presence of an open posterior glottic chink was noted if present. Adduction that occurred only during the second part of exhalation is a normal variant and was not recorded as PVFM.

The gold standard used for the diagnosis of PVFM during the study was a positive laryngoscopy demonstrating paradoxical vocal fold motion at baseline and/or post-HSC while symptomatic.

#### Analysis

All analyses were performed using statistical and data analysis software STATA (Statacorp, Texas, USA). Non parametric quantitative data were compared using the Wilcoxon rank sum test and for parametric data, ttest for matched pair data was used. Significance for 2 group comparison was set at p < 0.05.

## Results

**Figure 1:**
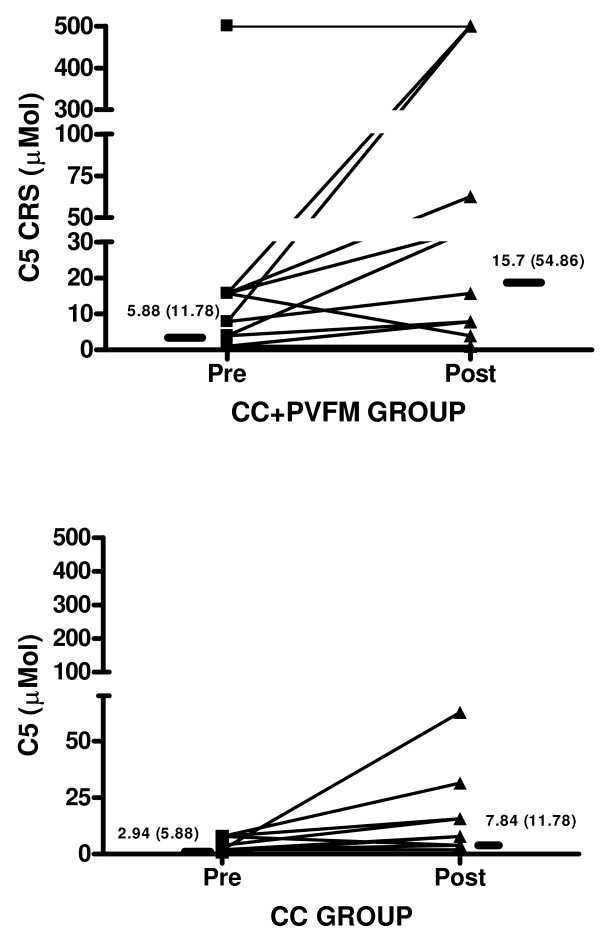
***a *Cough reflex sensitivity (CRS) to capsaicin before (pre) and after (post) treatment in the chronic cough with paradoxical vocal fold movement (CC+PVFM) group**. Solid bars are median values, with median (IQR) reported on figure, p = 0.005. C5 = capsaicin dose to elicit 5 or more coughs 30 sec after dose administered. *b *Cough reflex sensitivity (CRS) to capsaicin before (pre) and after (post) treatment in the chronic cough alone (CC) group. Solid bars are median values, with median (IQR) reported on figure, p = 0.04. C5= capsaicin dose to elicit 5 or more coughs 30 sec after dose administered.

**Figure 2:**
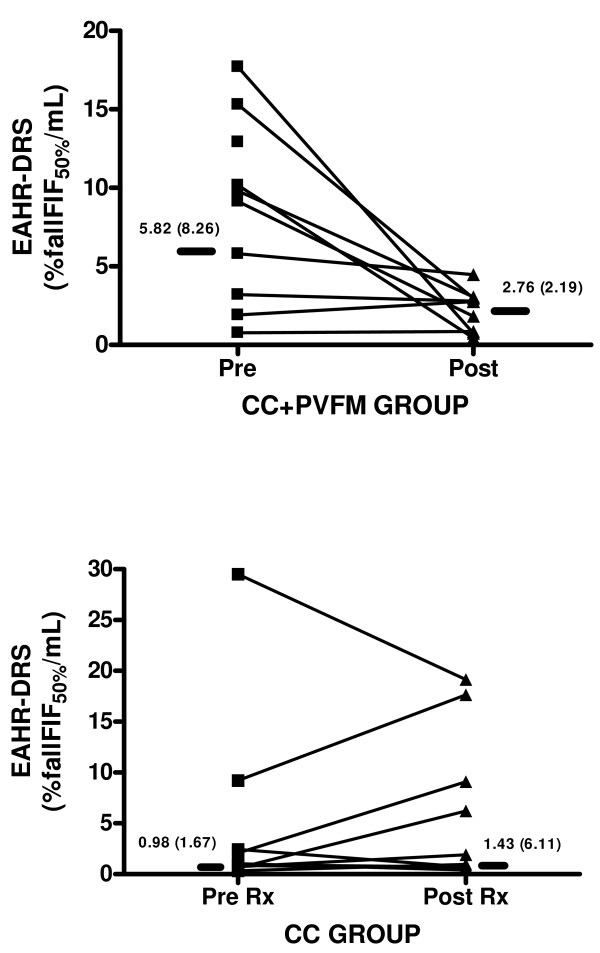
***a *Extrathoracic Airway Hyperresponsiveness (EAHR) represented as FIF_50 _Dose Response Slope to hypertonic saline provocation before (pre) and after (post) treatment in the chronic cough with paradoxical vocal fold movement (CC+PVFM) group**. Solid bars are median values, with median (IQR) reported on figure, p = 0.02. *b *Extrathoracic Airway Hyperresponsiveness (EAHR) represented as FIF_50 _Dose Response Slope to hypertonic saline provocation before (pre) and after (post) treatment in the chronic cough alone (CC) group. Solid bars are median values, with median (IQR) reported on figure, p = 0.58.

Twenty-four subjects with a chronic persistent cough participated in the study. The subjects had a median (IQR) cough duration of 24 (13–84) months and were predominantly female [Table 1]. There were 14 subjects with Cough+PVFM and 10 with Cough alone (CC). Subjects were treated [Table 2] and both groups responded with a significant improvement in cough-related quality of life (LCQ, p = 0.001 for Cough+PVFM Group, p = 0.01 for CC Group), associated diagnosis symptom questionnaire scores [Table 3] and cough reflex sensitivity (C5, p = 0.008 for Cough +PVFM Group and C5, p = 0.04 for CC Group), [Figures 1a, 1b]. For the Cough+PVFM subjects, we found that PVFM and extrathoracic airway hyperresponsiveness responded positively to treatment and was significantly reduced for the Cough+PVFM group, [Figure 2a] and unchanged for the CC alone group, [Figure 2b].

**Table 1:** Subject Characteristics. Median (IQR) unless otherwise stated.

	**Subject Characteristics**		
	**CC+PVFM**	**CC**	**P**

**Number**	14	10	

**Gender, M/F**	2/12	3/7	0.62

**Age, years**	56 (40)	58 (15)	0.88

**Age Range, years**	22–78	47–69	

**Exhaled CO, ppm Mean ± SEM**	1.69 ± 0.35	1.0 ± 0	0.10

**Cough Duration, months**	18 (48)	36 (168)	0.11

CC+PVFM = Chronic Cough + Paradoxical vocal fold movement

CC = Chronic Cough alone

**Table 2:** Subject Diagnosis and Treatment

**Diagnosis, n**	**CC+PVFM**	**CC**	**Treatment**
**Asthma**	7	5	Inhaled Corticosteroid

**GORD**	11	10	Proton Pump Inhibitor

**Rhinitis**	11 9	7 4	Nasal Steroid Antihistamine

**Eosinophilic Bronchitis**	1	3	Inhaled Corticosteroid

**Sleep Apnoea**	0	1	nCPAP

**PVFM**^†^	14*	0	Speech Language Therapy

*4 Subjects did not attend speech language therapy.

PVFM = paradoxical vocal fold movement

CC = chronic cough alone

CC+PVFM = Chronic Cough + Paradoxical vocal fold movement

nCPAp = nasal continuous airways pressure

**Table 3:** Change in symptom questionnaires before and after treatment. Median (IQR) unless otherwise stated.

	**CC+PVFM**			**CC**		
**Measurement**	**Baseline**	**Post Treatment**	**p**	**Baseline**	**Post Treatment**	**p**

**LCQ Score**	10.5 (3.1)	16.2 (1.5)	0.001*	10.4 (6.2)	17.5 (7.1)	0.01*

**GORD Score**	15 (7)	9 (6)	0.005*	15.5 (7)	11 (6)	0.02*

**Rhinitis Score**	9 (5.5)	4.5 (9)	0.04*	10.5 (3.5)	5 (6.5)	0.03*

**LDQ Score**	5 (4)	3.5 (4)	0.008*			

*A value of p < 0.05 considered to be significant

LCQ = Leicester cough questionnaire

GORD = Gastroesophageal reflux disease questionnaire

LDQ = Laryngeal dysfunction questionnaire

Ten of the 14 subjects with PVFM attended speech language therapy. After treatment, PVFM had resolved in 8 of these 10 subjects (p = 0.039 by McNemar's chi square test). Four of the Cough+PVFM subjects did not attend speech language therapy before returning for their post-treatment visits. PVFM did not resolve in 3 of these 4 subjects but did resolve in 1 subject. Interestingly this subject was the only male in this group of four and had the shortest cough duration (12 months) and youngest age (22 years).

In the Cough alone (CC) group, extrathoracic airway responsiveness was not increased and with therapy remained unchanged from baseline [Figure 2b]. Baseline spirometry and FENO were not altered by treatment for both cough groups [Table 4].

**Table 4:** Non-significant change in FENO and spirometry after cough treatment. Median (IQR) unless otherwise stated.

	**CC+PVFM**			**CC**		
	**Baseline**	**Post Treatment**	**p**	**Baseline**	**Post Treatment**	**p**

**FENO, ppb**	13.7 (8.8)	12.9 (7.6)	0.83	26.0 (18.9)	21.7 (13.2)	0.33

**FEV1 (%pred) Mean ± SEM**	90.8 (± 19.3)	90.7 (± 18.3)	0.48	90.8 (± 26.5)	91.9 (± 24.3)	0.54

**FVC (%pred)**	99.6 (29.1)	93.1 (15.0)	0.55	100.9 (15.9)	102.2 (17.4)	0.88

**FEV1/FVC (%)**	82 (8)	82 (11)	0.90	74 (9)	74 (10)	0.54

**FIF**_50%_**(L/s)**	2.97 (1.72)	2.85 (1.10)	0.38	4.07 (1.47)	4.08 (1.52)	0.11

**FIF**_50% _**(%pred)**	78.3 (± 30.0)	70.6 (± 24.4)	0.24	96.2 (± 36.5)	106.6 (± 31.3)	0.10

## Discussion

This study has identified that paradoxical vocal fold movement and extrathoracic airway hyperresponsiveness are improved by specific treatment for chronic persistent cough, and that this improvement occurs alongside improvements in cough specific quality of life and cough reflex sensitivity. The data provides objective evidence of laryngeal dysfunction in some patients with chronic cough, and shows that it responds to therapy for chronic persistent cough. These results are consistent with Vertigan et al [6] who found that a substantial proportion of their refractory chronic cough participants had extrathoracic airway hyperresponsiveness, similar to subjects who had vocal cord dysfunction (VCD), however they extend these results by showing that PVFM and EAHR can improve after treatment for chronic persistent cough.

Laryngeal dysfunction is increasingly recognized in chronic persistent cough. Symptoms such as voice hoarseness, dyspnoea, wheeze and cough may all occur as a result of laryngeal dysfunction [2]. Prudon et al have also reported laryngeal dysfunction in chronic cough where they described an enhanced glottic stop reflex in chronic cough patients [3]. These patients exhibited enhanced glottic closure in response to inhaled ammonia. Extrathoracic airway hyperresponsiveness is another manifestation of laryngeal dysfunction and has been reported in several conditions where cough is prominent, such as rhinosinusitis, ACE inhibitor cough, gastroesophageal reflux, and patients with asthma-like symptoms [4,5,25]. Speech language therapy is effective for laryngeal dysfunction, and it has previously been shown to be effective for refractory cough [6]. The results of the current study provide a mechanistic explanation for these responses by demonstrating that laryngeal dysfunction is responsive to treatment for chronic persistent cough, and correlates with an improvement in cough reflex sensitivity.

In this study we used an open design with objective measures to assess outcome. Although a nonrandomized design is a limitation, our primary purpose was to determine if the measures of laryngeal dysfunction that occur in chronic persistent cough are responsive to effective therapy. The study achieved these aims by using objective measures and has provided novel data on how PVFM and EAHR improve with therapy of chronic persistent cough. The results extend what is known about how successful therapy works in chronic persistent cough, and provide data that supports the favourable responses reported for symptoms, cough frequency, and measures of cough reflex sensitivity. We now show that laryngeal dysfunction also improves with treatment of chronic persistent cough in those patients with cough and PVFM. Future studies could provide further evidence of efficacy by using a randomized design, and potentially assessing any incremental benefits of speech language treatment.

We studied subjects who were representative of those with chronic persistent cough. They were primarily middle-aged females (80%) with a significant cough duration and similar prevalence of the medical conditions that have been associated with persistent cough [18,27,28]. We assessed cough reflex sensitivity to capsaicin using a validated technique and we found similar levels of cough reflex hypersensitivity to those reported elsewhere [18,28]. This suggests that the results can be generalized to patients with chronic persistent cough.

**Figure 3:**
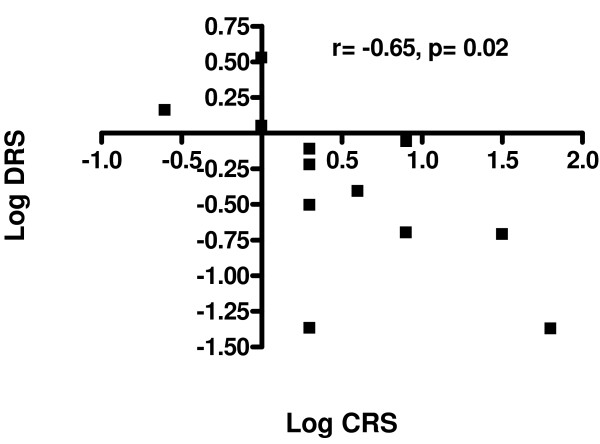
**Log change in Extrathoracic Airway Hyperresponsiveness (EAHR) represented as FIF_50 _Dose Response Slope (DRS) to hypertonic saline provocation correlated with log change in Cough Reflex Sensitivity (CRS) to capsaicin**.

There was a moderately significant (r = -0.65, p = 0.02) correlation in the Cough+PVFM Group for treatment related changes in extrathoracic airway hyperresponsiveness dose response slope and CRS-C5 [Figure 3]. This decrease in cough sensitivity corresponding with a fall in extrathoracic airway hyperresponsiveness dose response slope further supports validity of PVFM treatment with speech language therapy compared to no correlation between these two measures for the CC Group who did not undertake speech language therapy.

## Conclusion

In conclusion, this study identifies that the laryngeal dysfunction that occurs in some patients with chronic persistent cough is responsive to therapy.

## Text Abbreviations (In alphabetical order)

CC: Chronic Cough; CRS: Cough Reflex Sensitivity; EAHR: Extrathoracic Airway HyperResponsiveness; eCO: exhaled Carbon Monoxide; FENO: Forced Expired Nitric Oxide; FOL: Fibre Optic Laryngoscopy; GORD: GastroOesophageal Reflux Disease; HSC: Hypertonic Saline Challenge; IQR: InterQuartile Range; LCQ: Leicester Cough Questionnaire; LDQ: Laryngeal Dysfunction Questionnaire; PVFM: Paradoxical Vocal Fold Movement

## Competing interests

The authors declare that they have no competing interests.

## Authors' contributions

NR and PG planned the study. NR recruited the subjects and performed the objective cough and EAHR methods, questionnaires, assisted with fibreoptic laryngoscopy, collected and reviewed data, participated in the design and drafted the manuscript. PG performed patient assessment, physical examinations and fibreoptic laryngoscopy and prescribed medication. AV performed speech pathology treatment and reviewed the manuscript. PG also participated in the manuscript drafting and coordination of the manuscript. All authors read and approved the final manuscript.

## Sources of Funding

Nicole M Ryan holds a PhD scholarship from the NHMRC CCRE in Respiratory and Sleep Medicine, Australia.

Anne Vertigan holds a post-doctoral fellowship from the NHMRC CCRE in Respiratory and Sleep Medicine, Australia

Professor Peter Gibson is an NHMRC Practitioner Fellow.
